# Three Cases of Imperforate Hymen

**Published:** 1876-12

**Authors:** Samuel R. Burroughs


Three Cases of Imperforate Hymen.—*By Samuel R. Burroughs, M.D.*—Case I. In 1868, a negro man consulted me in reference to his infant child, a girl then three days old, whose condition he described as follows: The midwife, an aged negress, stated that the baby had passed no water from the bladder since its birth, but had had free dejections of a watery character from the bowels; that it was extremely restless, and would bend itself backward by sudden jerks and scream as if in great pain, and required almost constant nursing. Thinking there was some mistake in the matter, I gave it as my opinion that the urine probably passed when the bowels were moved, and directed a warm bath and a little spirits of nitre every three hours, and to closely examine the napkins. The next day the father returned, and stated that my directions had been followed, but no urine had been observed to pass; that the child had ceased to nurse, and was having convulsions. I now visited the patient, whom I found with convulsions recurring at intervals of twenty minutes, and between them crying and tossing incessantly. On examining the genitals, I discovered, slightly protruding between the labia, a thin, delicate membrane, containing minute blood-vessels, and somewhat distended by fluid. No meatus urinarius, or opening in the urethra, could be detected. Further examination revealed the membrane to be the hymen attached above to, or rather continuous with, the mucous membrane in front of the meatus urinarius, and completely blocking up the entrance both to the vagina and urethra. On introducing a delicate exploring needle, I discovered the confined fluid to be urine. The adjacent parts were much swollen from infiltration; a distinct and well rounded tumor was observable in the hypogastric region, over the whole of which there was distressing tenderness on pressure. A gentle tap with the finger over either the distended bladder or the protruding hymen revealed that the two contained the same fluid. Being satisfied as to the condition of things, I now enlarged the opening made by the exploring needle with a bistoury, when about a pint and a half of urine came away with a gush. I then proceeded to dissect up the entire membrane. But little hemorrhage occurred. I then introduced a pledget of lint well oiled, ordering it to be renewed as occasion required until the parts healed. The little patient soon after dropped to sleep. In four or five days the cut surfaces had cicatrized, and the case gave no further trouble.

Case II. Was in the person of a young lady, aged fifteen years, who was well formed and of good constitution. Her tongue was clean, and she had no fever. Yet she was the subject of a severe throbbing pain at the base of the occiput, grinding pains in the uterine region, radiating occasionally to the loins and inside of the thighs, intermittent in character, and appearing with a certain degree of regularity, simulating somewhat the advent of a real labor. Examination of the abdomen revealed a pear-shaped tumor, the summit of which reached nearly to the umbilicus. The tumor was firm, though somewhat elastic. When pressed upon, the most excruciating pains were excited in the lower extremity of the tumor, or in that part corresponding to the os uteri.

The most carefully conducted examination failed to discover fluctuation or a placental souffle, or the sound of a fœtal heart. The mother said that the patient had never menstruated, and had enjoyed excellent health until six months before, when she complained of dull pains in the head, back and hypogastrium, accompanied by anorexia and slight nausea, a disposition to sleep, and an indisposition to either physical or mental exertion. These symptoms, which usually recurred every twenty-five to thirty days, generally reached their height on the third day, and then gradually subsided in about as many more, fair health, or at least comfort, being at first the rule during the interim. But as they were repeated they became more and more severe, until the general health was finally disturbed. No other than domestic remedies had been used in the case. I proposed a digital examination, which was refused. I then gave a dose of chloral-hydrate, which secured a pleasant night's sleep. The pains gradually ceased, and the molimen slowly passed off.

At the end of a month I was called in great haste to see the patient, and found her suffering pains similar to and as great as those of labor. She was in tears, and on the accession of the pains would scream at the top of her voice. There were cramps in the calves of the legs, and sharp transient pains in the mammæ, which were somewhat enlarged and hard. The pulse was a little accelerated, the tongue coated, and the temperature slightly increased. There was frequent desire to micturate, the act being attended by scalding, while only a few drops of urine escaped.

A vaginal examination was now no longer opposed, and led to the discovery of a scarlet red, fluctuating tumor, the size of a goose-egg, lying between the labia majora. The catheter brought away a pint of highly-colored urine. Fluctuation in the tumor was easily detected, both in that part of it which lay between the labia and that which occupied the hypogastric region, and a light blow in either situation was communicated to the opposite extremity of the swelling. An examination by the rectum detected the presence of a fluid in the vagina. Being now fully satisfied as to the nature of the case, I plunged a bistoury through the walls of the projecting cyst, when a black, grumous-looking fluid escaped with a force which projected it a distance of five or six feet. The patient experienced almost instantaneous relief. The total amount of fluid discharged at the time was estimated at twenty-five or thirty ounces, but it continued to pass in clots and small quantities for several days longer. As soon as the main flow had ceased, I made a second incision, transverse to the first, through the entire extent of the membrane, introduced an oiled tent, and enjoined attention to cleanliness. The patient rapidly recovered her health, and had no further trouble.

Case III. A lady stated that her daughter, who was nearly fourteen years of age, had been in good health until five months before, when she suffered for several days with pain in the head and back, loss of appetite and strength. She furthermore said that these phenomena had since that time returned at regular monthly epochs, with some increase in severity at each successive period; that she had never menstruated, and at that time was suffering much from pain in the lower part of the abdomen and back, the former being swollen and rather tender to the touch.

Being unable to visit the patient at the time, and supposing that the symptoms arose from other than a local cause, I directed iron and quinia, warm clothing, out-door exercise, good food, etc. Two months subsequent to this time the girl's mother brought her to my office for examination. An abdominal tumor was as manifest as in Case II. It really simulated a uterus gravid six months. A dense, unyielding, imperforate hymen fluctuating through it, and also through the rectum, and most of the other features noted in Case II, go to complete the picture. The next day I operated at the patient's residence by a crucial incision, giving exit to twenty or thirty ounces of black, decomposed blood, intermingled with purulent matter. After the greater part of this liquid mass had passed, I dissected the four quarters of the disk from their original continuity, and placed an oiled tent in the opening, and directed that with the necessary renewals it should be kept *in situ* until the parts healed. For an hour or two after the operation the girl suffered considerably with uterine pains, which, however, yielded to chloral hydrate, and under proper regimen her health was speedily regained. The membrane removed in this case was about one-eighth of an inch in thickness, very dense in structure, and sufficiently rigid to prevent the pressure of the retained fluid from causing it to bulge or protrude externally.

In conclusion, I may be permitted to remark that Case I is not only interesting from its rare occurrence, but in that it combines both a malformation and a malposition, converting the genito-urinary apparatus into one irregular common canal. I have failed so far to find a record of a similar case in the books and journals to which I have had access.

Cases II and III are important inasmuch as they establish the following facts: First, they show the necessity of carefully examining the genito-urinary organs of all children born under our professional care, and correcting, if possible, such deformity as may be discovered. Second, that great suffering, constitutional disturbance, and much embarrassment both on the part of the patient and medical attendant, may obtain in these cases from a neglect of the above precaution. Finally, it is also worthy of remark that neither of these cases exhibited any symptoms whatever that could be attributed to the results of a regurgitation of the retained liquid through the fallopian tubes, although uterine contractions were excited by increasing volume and continued presence.—*Am. Prac.*

